# A rapid liquid biopsy of lung cancer by separation and detection of exfoliated tumor cells from bronchoalveolar lavage fluid with a dual-layer “PERFECT” filter system

**DOI:** 10.7150/thno.44274

**Published:** 2020-05-17

**Authors:** Tingyu Li, Yaoping Liu, Wei Zhang, Lianjun Lin, Jixin Zhang, Yan Xiong, Ligong Nie, Xinmin Liu, Haichao Li, Wei Wang

**Affiliations:** 1 Institute of Microelectronics, Peking University, Beijing, 100871, China;; 2 Antimicrobial Resistance (AMR) and Critical Analytics for Manufacturing Personalized-Medicine (CAMP) IRG, Singapore-MIT Alliance for Research and Technology (SMART) Centre, Singapore, 138602, Singapore;; 3 Department of Respiratory and Critical Care Medicine, Peking University First Hospital, Beijing, 100034, China;; 4 Department of Geriatrics, Peking University First Hospital, Beijing, 100034, China;; 5 Department of Pathology, Peking University First Hospital, Beijing, 100034, China;; 6 National Key Laboratory of Science and Technology on Micro/Nano Fabrication, Beijing, 100871, China;; 7 Frontiers Science Center for Nano-optoelectronics, Peking University, Beijing, 100871, China.

**Keywords:** lung cancer, liquid biopsy, exfoliated tumor cells (ETCs), bronchoalveolar lavage fluid (BALF), PERFECT filter

## Abstract

Separation and detection of exfoliated tumor cells (ETCs) from bronchoalveolar lavage fluid (BALF), namely the liquid biopsy of BALF, has been proved to be a valuable tool for the diagnosis of lung cancer. Herein, we established a rapid liquid biopsy of BALF based on a dual-layer PERFECT (precise, efficient, rapid, flexible, easy-to-operate, controllable and thin) filter system for the first time.

**Methods**: The dual-layer PERFECT filter system consists of an upper-layer filter with large micropores (feature size of 49.4 ± 0.5 μm) and a lower-layer filter with small micropores (9.1 ± 0.1 μm). The upper-layer filter contributes to the isolation of cell clusters and removal of mucus from BALF samples, meanwhile the lower-layer one targets for the separation of single ETCs. First, separation of 10000 spiked A549s (cultured lung cancer cells) from 10 mL clinical BALF samples (n=3) were performed to investigate the performance of the proposed system in rare cell separation. Furthermore, separation and detection of ETCs and ETC clusters from clinical BALF samples were performed with this system to test its efficacy and compared with the routine cytocentrifuge. The clinical BALF samples were collected from 33 lung cancer-suspected patients with visible lesions under bronchoscope. The final histopathological results showed that 20 samples were from lung cancer positive patients while the other 13 cases were from lung cancer negative patients.

**Results**: The recovery rate of spiked A549 cells from clinical BALF samples with the developed system (89.8 ± 5.2%) is significantly higher than that with the cytocentrifuge (13.6 ± 7.8%). In the preliminary clinical trial, although 33 clinical BALF samples with volume ranging from 6 mL to 18 mL showed greatly varied turbidity, filtrations could be finished within 3 min for 54.6% of samples (18/33), and 10 min at most for the rest. The dual-layer PERFECT filter system is proved to have a much higher sensitivity (80.0%, 95% CI: 55.7%-93.4%) than the routine cytocentrifuge (45.0%, 95% CI: 23.8%-68.0%), *p*=0.016 (McNemar test, two-tail). Moreover, the sensitivity of this platform is neither interfered by the variations of turbidity of the BALF samples, nor associated with the types of lung cancer.

**Conclusions**: The easy and rapid processing of BALF samples with varying volume and turbidity, competitive sensitivity and good versatility for different lung cancer types will make the established dual-layer PERFECT filter system a promising approach for the liquid biopsy of BALF. The high-performance BALF-based liquid biopsy will improve the cytopathological identification and diagnosis of lung cancer.

## Introduction

Lung cancer is the deadliest malignancy with high incidence rates (11.6% worldwide, 13% in USA, 17% in China) and high mortality rates (18.4% worldwide, 23-24% in USA, 22% in China) among all kinds of cancers all over the world 1-3. Five-year relative survival rate is only 5% for patients with lung cancer diagnosed at the advanced stage, while it significantly increases to 56% for those diagnosed at the early stage 2. Therefore, early identification and diagnosis along with timely treatment has been acknowledged to be critical to improve the patient outcomes.

The present widely-used approaches for lung cancer diagnosis 4, 5 mainly include imaging screening and biopsy. Imaging methods, such as plain chest X ray, computerized tomography (CT) and positron emission tomography (PET), have obvious advantages of easy operation and no/low invasion. However, they are suffering a high false positive rate. For example, the false positive rate of low-dose computerized tomography (LDCT), a screening approach for high-risk lung cancer populations recommended by American Thoracic Society and American College of Chest Physicians (ATS and ACCP) 6, is as high as 96.4% 7, 8, which may lead to unnecessary follow-up examinations and cause anxiety and panic to patients. Sampling from lesions and conducting pathological examinations, is the only standard for the final identification and diagnosis of lung cancer in clinical practices. Pathology can be divided into histopathology and cytopathology according to the types of examined specimens (tissues and cells), which correspond to tissue biopsy and liquid biopsy, respectively. Histopathology has been conventionally used as the gold standard, considering adequate tumor contents can be obtained in the tissue biopsy via surgery or imaging-guided needling, and thereby can provide more information for identification of tumor stage and grade. However, the sampling procedures are more invasive and riskier to complications, and even unavailable for those in poor physical condition. For liquid biopsy, although the sensitivity and specificity of cytological examination are variable among different specimen sources and different handling methods, the benefits of liquid biopsy including minimal invasiveness, rapidness and ease of repeatable tests are clear in terms of patient acceptability and disease management. Liquid biopsies can complement the tissue biopsy, allowing more patients to be tested, and thus are worth exploring. Extensive attentions and efforts have been devoted to developing advanced strategies to promote applications of liquid biopsy in clinical practices during the last decade 9-12.

As for the liquid biopsy of lung cancer, besides the recent mainstream blood-based test, the cytological examination of bronchoalveolar lavage fluid (BALF) has been an adopted diagnostic method for lung cancer in hospitals since 1980s 13, 14. However, the presently used cytological examination approaches suffer a low detection sensitivity, which degrades the application value of BALF in lung cancer diagnosis. The conventional approaches can be mainly classified into two types: centrifugation-based and filtration-based methods, with SurePath and CYTOfast, track-etched membrane and Thinprep as typical commercial products, respectively 15. The centrifugation-based methods are the most commonly used methods for cell recovery from liquid sample but always result in considerable cell loss. The filtration-based methods are applied via separating the larger target cells from smaller background cells. However, the filters embedded in the existing commercial products are of low porosity, non-uniform pore size and non-uniform pore-to-pore space, which lead to easily-happened clogging (*i.e.* limited volume sample to be operated) and insufficient cell recovery ability. The previously reported diagnostic sensitivities of BALF for lung cancer based on the conventional methods range from 7% (peripheral lesions < 2 cm) to 50% (central lesions > 3 cm). Clearly, the sensitivity and diagnostic efficacy of BALF was influenced by the size and segmental location of the lesion 4, 16-19. The low sensitivities are attributed to two factors: the cell loss and the limited sample volume operation capability by the aforementioned methodologies for exfoliated tumor cells (ETCs) separation and detection from BALF. Therefore, to promote the use of BALF-based liquid biopsy of in the clinical diagnosis of lung cancer, the key is increasing the separation efficiency and detection sensitivity of rare ETCs at a high operation throughput from large-volume samples.

In recent years, with the involvement of micro/nanotechnology, the capability of rare cell recovery from clinical fluids has been significantly improved. Various liquid biopsy techniques have been reported, including microfluidics-based (cell-affinity micro-chromatography 20-23, hydrodynamic microfluidics 24-26, other cell physical properties 27-29, *etc*.) and filtration-based techniques (micropillar 20, 30, microweir 31, micropore 32-34). The filtration-based liquid biopsy has been well acknowledged to be promising for achieving a high throughput and thus handling large-volume clinical samples. A lot of micropore-arrayed filters 32-34 with uniform pore size and pore-to-pore space have been achieved with advanced micro/nano fabrications and have realized high-efficiency cell recovery during the past decade. However, the porosities of those filters are still low and only realize a filtration throughput not higher than 2 mL/min, which cannot fulfill the process of large-volume BALF samples, although is sufficient for liquid biopsy of blood. A high-porosity PERFECT (precise, efficient, rapid, flexible, easy-to-operate, controllable and thin) filter for efficient rare cell separation from large-volume samples at a high throughput (>110 mL/min for aqueous sample, >17 mL/min for undiluted whole blood, only driven by the gravity) has been established in our previous work 35. And a high recovery rate of 86.0 ± 5.3% for the detection of ~10 spiked cultured lung cancer cells (A549s) from 10 mL non-cancer BALF samples with a PERFECT filter with micropores of feature size at 9.1 ± 0.1 μm (Filter-1 as listed in Table 1) has been verified. However, BALF samples from lung cancer patients are more complex with contents of mucus, ETCs, ETC clusters, epithelial cells, leukocytes, erythrocytes, *etc*. Clogging problem exists in the single-layer PERFECT filtration of most BALF samples. In addition, the ETC clusters are thought to be more metastatic and of important value for tumor monitoring, and worth efforts to separate and detect, which was not involved in our previously preliminary work. This work proposes a dual-layer PERFECT filter system for ETC (cluster) separation and detection from raw BALF samples. Two filters with different feature sizes are assembled into the working system. The upper-layer filter contains larger micropores (feature size at 49.4 ± 0.5 μm, Filter-3 as listed in Table 1) for the separation of ETC clusters and removal of mucus, while the lower-layer one contains smaller micropores (9.1 ± 0.1 μm, Filter-1 as listed in Table 1) for the separation of rare single ETCs. The choice of upper-layer feature size is systematically investigated and optimized in this work, and the decision of lower-layer feature size is according to our previous work verifying efficient recovery of rarely spiked A549 from BALF samples 35. Moreover, to demonstrate the efficacy of the developed dual-layer PERFECT filter system, a preliminary clinical trial containing 33 BALF samples from patients with visible lesions under bronchoscope was performed, with the routine approach as a parallel comparison.

## Methods

### Set-up of dual-layer PERFECT filter system

**Figure 1:**
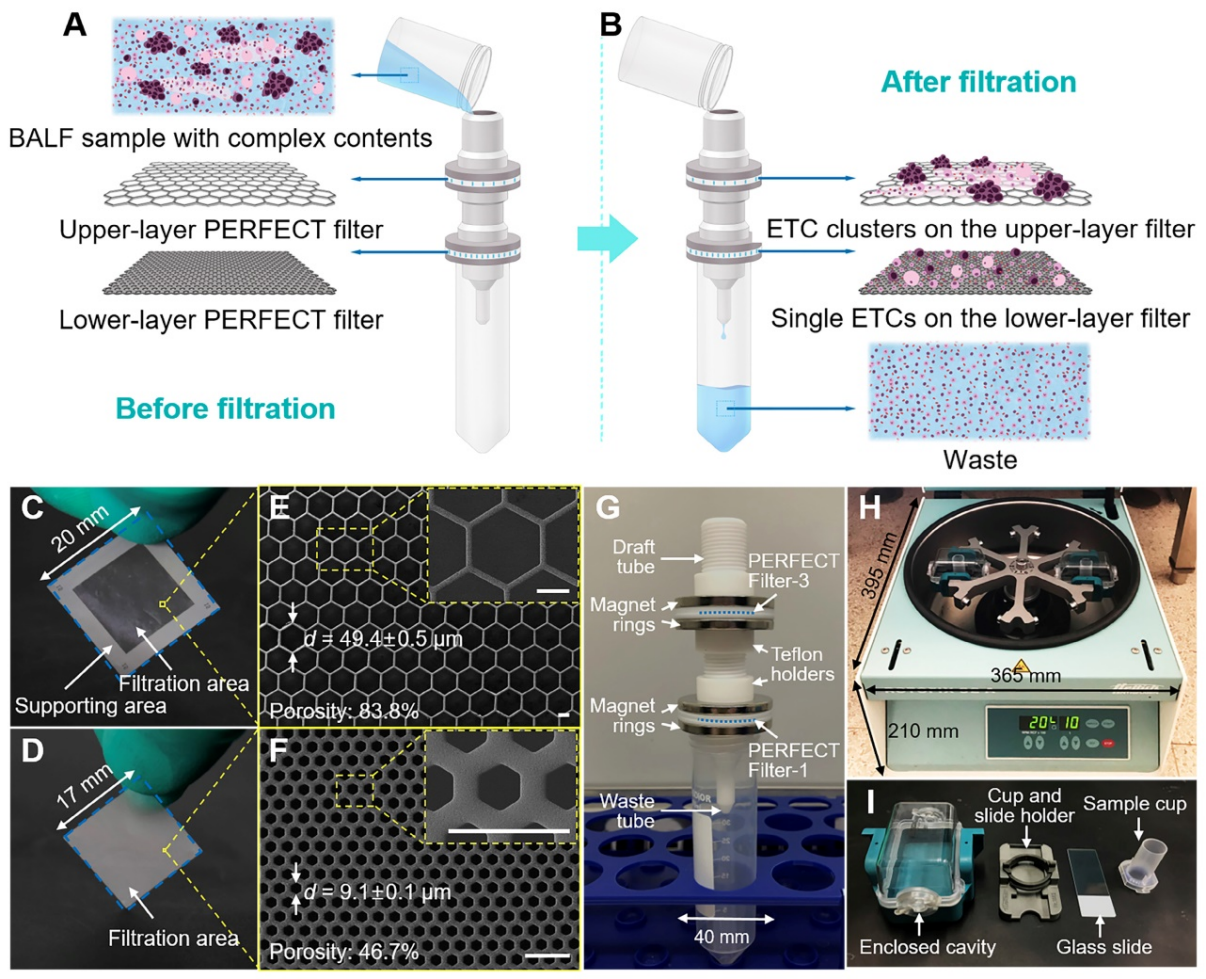
(A, B) Schematic illustration of the working principle of the present dual-layer PERFECT filter system for the ETC (cluster) separation from BALF samples. (C, D) The photos and (E, F) SEM images of the upper-layer filter with large micropores and lower-layer filter with small micropores (Filter-3 and Filter-1 as listed in Table 1, respectively). Scale bars represent 20 μm in E, F. (G) The assembled dual-layer filter system, with home-designed Teflon gadgets and ring magnets for interconnection and sealing. (H, I) The cytocentrifuge used as a parallel comparison in this work.

The BALF samples are of complex contents, including mucus, ETCs, ETC clusters, epithelial cells, leukocytes and erythrocytes. A dual-layer filtration system is proposed here for effective ETC (cluster) separation from raw BALF samples, schematically shown in Figure 1A-B. The ETC clusters and mucus are collected on the upper-layer filter. The single ETCs, large epithelial cells and leukocytes are captured on the lower-layer filter. The small epithelial cells, leukocytes and erythrocytes together with the fluid passed through the filters and are collected in the waste tube.

To decide the feature size of the upper-layer filter, filers with different micropore diameters (Filter-2, -3 and -4 as listed in Table 1) were experimentally investigated to optimize the filtration performance. The selection of lower-layer filter (Filter-1 as listed in Table 1) is from the optimized results of our previous related work 35, for the efficient recovery of single ETCs. The feature sizes, including the diagonal length (*d*) and space between adjacent micropores (*s*), and porosities (*p*) of PERFECT filters used in this work are listed in Table 1.

The PERFECT filters were fabricated with our previously reported Parylene C molding technique 36. The photos and typical scanning electron microscopy (SEM) images of the upper-layer filter and lower-layer filter are displayed in Figure 1C, E and D, F, respectively. As shown in Figure 1G, the upper-layer filter and lower-layer filter were sequentially packaged from top to bottom and assembled into the working system with the home-designed Teflon gadgets, for filtration of BALF samples. The sealing of filters, clamp of Teflon gadgets, was simply but effectively achieved with two ring magnets.

### The routine cytological test currently used in hospitals

All clinical BALF samples were processed in parallel with a hospital-used cytocentrifuge for liquid-based cytology as comparison. The cytocentrifuge system used in this study, as displayed in Figure 1H-I, was purchased from Hettich (ROTOFIX32A), Germany. Briefly, the BALF sample with according volume for each case was loaded into the sample cup, as shown in Figure 1I, with an upper limit of 10 mL, which was decided by the cell density predicted with the nephelometric reading, to guarantee a monolayer of cells for the convenience of observation under microscopy and accuracy of cytological recognition. Centrifugation at 2000 rpm for 10 min was applied, and the supernatant was discarded, followed by staining and reading of the collected cells on the slides.

### Patient inclusion criteria

The inclusion criteria of clinical cases in the present work were that the patients were suspected of having lung cancer and reached the requirement of bronchoscopy test, plus with visible lesions under bronchoscope. From November, 2017 to June, 2018, 33 patients who were highly suspected as lung cancer and required bronchoscopy evaluation were enrolled in this study in the Peking University First Hospital. As a blind test, the patient status (having malignant or benign tumor) was not disclosed to the operators during the BALF sample collection, process and pathological assessment. The final histopathological diagnosis results revealed that, 20 cases are malignant tumors while the other 13 cases are benign tumors.

### Collection and preservation of BALF samples

Collection of BALF samples was approved by the ethics board committees and signed by the chairman of the committee of in the Peking University First Hospital (Approved No.: 2017-1385). Informed consents were obtained from patients for all the experimentations.

The BALF samples were collected during the operation of bronchoalveolar lavage (BAL) performed in the lesion area of interest, in compliance with the relevant laws and institutional guidelines (Good Clinical Practice (GCP) of China). BAL was performed under general anesthesia. After a thorough inspection of the airway, warm normal saline was instilled into the target segment and collected via suction. The obtained BALF samples (about 12-36 mL) were stored in 4 ℃ and processed within 6 h with the present dual-layer PERFECT filter system and the cytocentrifuge in parallel. Tissue biopsies were also performed. Final diagnoses of malignancies were confirmed by histopathological results.

### Verification of ETC separation with spiking BALF samples

To verify the recovery rate of this dual-layer PERFECT filter system, spiking BALF samples were first tested. The spiking samples were prepared by adding 10000 A549 cells (purchased from the National Infrastructure of Cell Line Resource, Beijing, China) via serial dilutions into 10 mL BALF samples. A549s were pre-labeled with Cell Tracker Green (5 μM, C2925, Invitrogen™, Thermo Fisher, USA) and Hoechst 33342 (5 μg/mL, H1399, Invitrogen™, Thermo Fisher, USA), in order to facilitate the observation and counting of the recovered A549s among background cells from BALF under the fluorescence microscope (CKX53, Olympus, Japan). Separation of A549 cells from the spiking samples were carried out via the dual-layer PERFECT filter system, single-layer PERFECT filter and the cytocentrifuge, in parallel. After filtration and centrifugation, the separated cells on filters were mounted between glass slides and cover slips with the Antifade Mountant (P36961, Invitrogen™, Thermo Fisher, USA), observed and counted under a fluorescence microscope to get the recovery rates. All experiments were repeated for three times.

### Separation and detection of ETCs (clusters) from raw BALF samples

The obtained BALF samples from lung cancer-suspected patients were performed via both the present dual-layer PERFECT filter system and the cytocentrifuge. Loading volume and filtration time for each sample was recorded. The collected cells on filters and glass slides were air dried and fixed with 95% ethanol for 10 min, followed by the hematoxylin-eosin (HE) staining. The procedure of HE staining included sequential immersions in hematoxylin (CS700, Dako, USA) for 2-3 min, bluing buffer (CS702, Dako, USA) for 10-15 s and eosin (CS701, Dako, USA) for 10-15 s. Rinse with distilled water was conducted after each immersion. Then they were dehydrated with 70% ethanol, 95% ethanol, absolute ethanol and dimethylbenzene sequentially, and mounted with neutral balsam for long-term stable storage.

### Cytopathological assessment of separated cells from BALF

HE stained cells on filters and glass slides were observed and double-interpreted by two experienced pathologists from the department of pathology, Peking University First Hospital, according to cytological guidelines 19, 37, 38.

### Statistical analysis

The cytopathological results from both the present dual-layer PERFECT filter system and the cytocentrifuge were compared with the routinely used gold standard, histopathological results, to verify the sensitivity and specificity. The 95% confidence intervals (95% CI) for sensitivity were calculated. McNemar test was used to compare the disagreement of sensitivities between the dual-layer PERFECT filter system and the cytocentrifuge in the cytological diagnosis of BALF. Kappa test was carried out to evaluate the agreement between the histopathological diagnosis and BALF-based cytological diagnosis, including the dual-layer filtration-based and cytocentrifuge-based approaches. Statistical analyses were performed using VassarStats: Website for Statistical Computation 39.

## Results and Discussion

### Optimization of the dual-layer PERFECT filter system with spiking BALF samples

**Figure 2:**
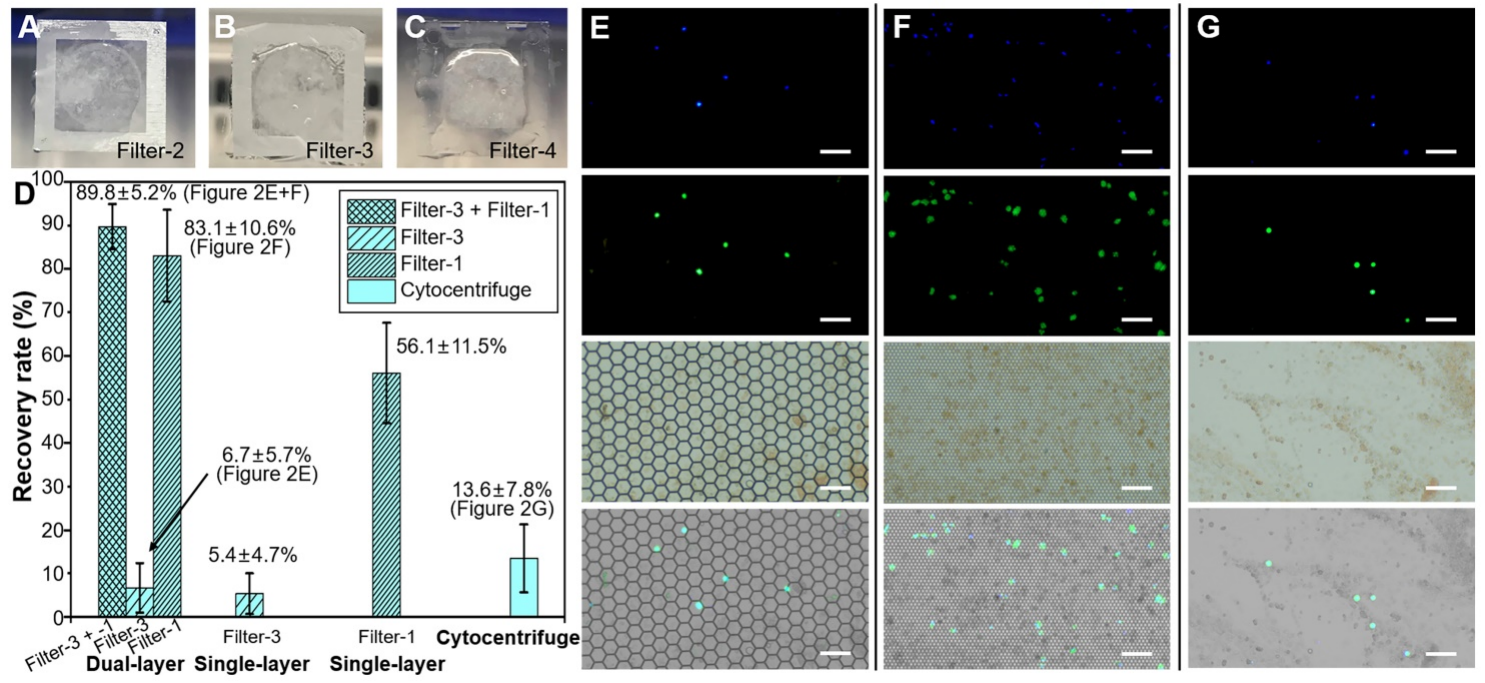
(A-C) The photos of PERFECT filter-2, -3 and -4 after filtration of BALF. (D) The recovery rates of 10000 pre-labeled A549 cells spiked in 10 mL BALF with the dual-layer (Filter-3 and Filter-1, sequentially), single-layer (Filter-3 only), single-layer (Filter-1 only) and cytocentrifuge, respectively. (E-F) The images of separated A549 cells on the Filter-3 and Filter-1 through the dual-layer filtration. (G) The images of separated A549 cells on the glass slide after centrifugation. Scale bars represent 100 μm in E-G.

Filter-1 was used as the lower layer because of its verified high recovery rate in our previous work 35 for the recovery of rarely spiked A549s from 10 mL clinical BALF samples. In the previous work, the non-cancer BALF samples were of clear appearances with few mucus. However, BALF samples from lung cancer patients were more complex and easily to form clogging if only filtered with the Filter-1, which led to a low throughput and a low detection sensitivity. To address this issue, hereby a dual-layer PERFECT filter system was designed, which consists of an upper-layer and a lower-layer PERFECT filter for the removal of mucus and isolation of cell clusters, and the recovery of single ETCs, respectively. To optimize the feature size of upper-layer PERFECT filter, a BALF sample was divided in to three aliquots, and filtered through three different filters (Filter-2, -3 and -4). The filtration results are shown in Figure 2A-C. Filter-2 with micropores of feature size at 24.5 ± 0.1 μm and porosity at 71.5% still suffered the mucus blocking issue, leading to a low throughput. Filter-4 with micropores of feature size at 99.4 ± 0.5 μm and porosity at 91.4% is of a low mechanical strength, and was easily deformed even when the filter was only partially clogged. Filter-3 with micropores of feature size at 49.4 ± 0.5 μm and porosity at 83.8% showed a good compromise between the throughput and mechanical strength for the filtration of raw BALF samples with complex contents. Therefore, the following dual-layer PERFECT filter system used for clinical trial was implemented with Filter-3 and Filter-1 as the upper layer and lower layer, respectively.

Recovery results of spiked A549 cells (10000) from BALF samples with dual-layer (Filter-3 and Filter-1, sequentially), single-layer (Filter-3 only and Filter-1 only) and cytocentrifuge were compared in Figure 2D. The dual-layer PERFECT filter system (Filter-3 + Filter-1) achieved a high recovery rate of 89.8 ± 5.2% (i.e. the ratio of the number of recovered A549 cells on the Filter-3 plus that on the Filter-1 to the total number of spiked A549 cells), with 6.7 ± 5.7% and 83.1 ± 10.6% of spiked A549s recovered on the Filter-3 and Filter-1, respectively. Typical fluorescent images of separated A549s on Filter-3 and Filter-1 through dual-layer filtration are shown in Figure 2E-F. The diameter of A549 is 12.8 ± 2.5 μm from our measurements, and it is much smaller than the feature size of micropores in Filter-3 (diagonal length of 49.4 ± 0.5 μm), while larger than that of Filter-1 (diagonal length of 9.1 ± 0.1 μm). Therefore, 83.1 ± 10.6% of the spiked single A549 cells passed through the Filter-3 and were subsequently recovered on the Filter-1. There were 6.7 ± 5.7% A549 cells recovered on the Filter-3 because of the adhesion onto the large-sized mucus. The single-layer filtration through Filter-3 only presented a low recovery rate of 5.4 ± 4.7%, similar to that of the upper-layer Filter-3 in the dual-layer PERFECT filter system. Meanwhile, the single-layer filtration through Filter-1 had a recovery rate of 56.1 ± 11.5%, which was much lower than that of the Filter-1 used as the lower layer in the dual-layer PERFECT filter system. It is because that mucus in the BALF samples easily blocked most area of the Filter-1, and failed the operation of the whole sample, and thus caused most of A549 cells to be lost with the disposed supernatant. The dual-layer PERFECT filter system presents a dramatically improved performance in the recovery of rare targeted cells from BALF samples, compared to the single-layer filtrations. However, the cytocentrifuge as the comparison presents a low recovery rate of 13.6 ± 7.8% (Figure 2G). The low recovery rate of the cytocentrifuge was attributed to that most of A549 cells were discarded with the supernatant after centrifugation. The results indicated that the proposed dual-layer PERFECT filter system fulfills a significantly higher recovery rate of A549 cells than the traditionally used cytocentrifuge, and thereby holds promising potential to improve the sensitivity of the diagnosis of lung cancer with BALF as specimens.

### Separation and detection of ETCs (clusters) from raw BALF samples

#### High filtration throughput of the dual-layer PERFECT filter system

**Figure 3:**
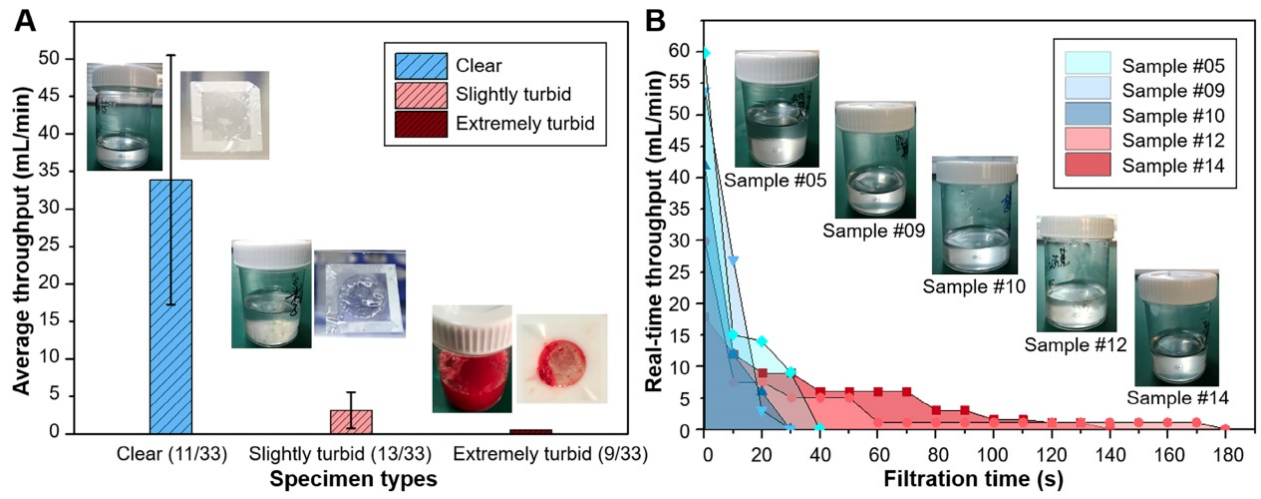
(A) Average filtration throughputs of the three graded BALF samples with different turbidity with the dual-layer PERFECT filter system. Insets showed the photos of typical BALF samples before filtration and the Filter-3 after filtration to give a visual sense of the differently graded turbidity. (B) Real-time throughputs of the typical clear BALF samples (#05, #09 and #10) and slightly turbid BALF samples (#12 and #14). Insets showed the appearance of these typical BALF samples.

33 patients with visible lesions under bronchoscope were enrolled in the preliminary clinical trial of this study, with the detailed information of patients listed in Table 2. The BALF samples collected from patients were of great variances, especially in terms of turbidity (the content percentage of mucus) and volume, which might strongly influence the filtration performance. In this work, the collected BALF samples with volume of 12-36 mL were divided into two aliquots, with one processed with the dual-layer filtration system and the other one with the conventionally used cytocentrifuge. The turbidity of the 33 samples was classified into three grades: clear (+) for 11 samples, slightly turbid (++) for 13 samples, and extremely turbid (+++) for 9 samples, according to the mucus content percentages from the appearance, with typical photos of different turbidity grades displayed in the insets of Figure 3A.

All the BALF samples with volume of 6-18 mL can be operated within 10 min with the dual-layer PERFECT filter system. It enables a short time-to-result (<30 min) from obtaining the sample to reporting the diagnosis result, together with the HE staining (~10 min) and manual cytological interpretation (~10 min). Average throughputs for clear (31.9 ± 17.3 mL/min), slightly turbid (3.2 ± 2.6 mL/min) and extremely turbid (<0.5 mL/min) BALF samples were shown in Figure 3A. The results indicated that the lower turbidity of BALF, the higher throughput. Clear (11/33) and slightly turbid (13/33) BALF samples were filtrated smoothly and completely. Among them, 54.6% BALF specimens (18/33) were filtrated within 3 min, and others could be finished completely within 10 min. However, clogging by mucus still happened in the extremely turbid BALF samples (9/33), which hindered the whole samples going through the filters. For these cases, the filtrations were stopped by discarding the unfiltered sample when it consumed up to 10 min (filtration time was recorded as >600 s in Table 2). Even so, our system still can recover sufficient cells for diagnosis, which will be further discussed in the following sections. Typical photos of the three graded BALF samples before filtration and the appearance of Filter-3 after filtration were shown as the insets in Figure 3A.

Figure 3B shows the real-time throughputs of three clear and two slightly turbid BALF samples. All the five samples showed high throughputs at the beginning, and throughputs gradually decreased along with the mucus blocking and cell accumulating on the PERFECT filters. Pretreatment of digestion to remove mucus in the turbid BALF samples could be an option to facilitate an improved filtration throughput, and will be investigated and optimized in future.

#### High detection sensitivity and specificity of the dual-layer PERFECT filter system

**Figure 4:**
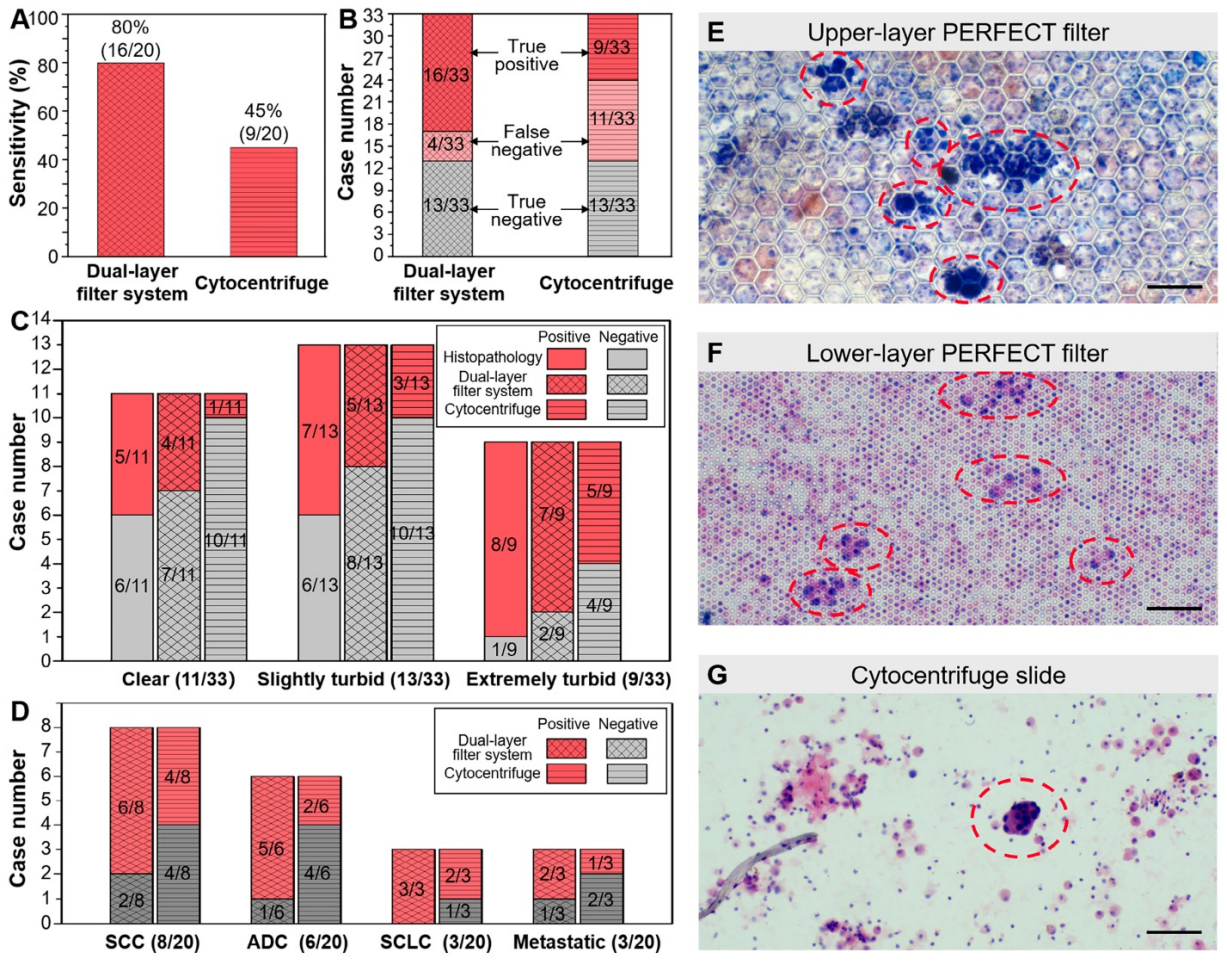
(A) Higher sensitivity of the established dual-layer PERFECT filter system (80.0%) than that of the cytocentrifuge (45.0%). (B) The specifically lung cancer positive and negative case numbers of all 33 samples with the dual-layer PERFECT filter system and the cytocentrifuge. (C) The sensitivities of all the three graded turbidity samples with the dual-layer PERFECT filter system are higher than those with cytocentrifuge. (D) The sensitivities of all the lung cancer types with the dual-layer PERFECT filter system are higher than those with the cytocentrifuge. (E-G) The ETC clusters and ETCs circled with red dashed lines on the upper-layer PERFECT filer, lower-layer PERFECT filer and the slide after centrifugation. Scale bars represent 100 μm in E-G.

Among the enrolled 33 patients, 20 cases were finally diagnosed as lung cancer positive while the other 13 patients were negative according to histopathological results. As shown in Figure 4A, the sensitivity of the present dual-layer PERFECT filter system is 80.0% (16/20) with 95% CI of 55.7%-93.4%, while that of the cytocentrifuge is only 45.0% (9/20) with 95% CI of 23.8%-68.0%. In the McNemar test, the calculated *p*=0.016 (two-tail) indicates that the BALF diagnosis using the dual-layer PERFECT filter system is significantly advantageous than using the cytocentrifuge. All the lung cancer negative BALF samples were all correctly diagnosed by the two approaches, presenting a high specificity of 100%. As shown in Figure 4B, the dual-layer PERFECT filter system presents false negative results in 4 cases, which is significantly lower than that from the cytocentrifuge (11 cases), for the samples (20 cases) diagnosed to be lung cancer positive according to the histopathological results. The Kappa test shows the dual-layer PERFECT filter system (*κ*=0.759) has better agreement than the cytocentrifuge (*κ*=0.392), compared with the histopathological diagnosis.

The key for high detection sensitivity of the developed dual-layer filtration system in the separation and detection of ETCs from large-volume BALF samples is that it can fulfill a high throughput of sample operation and a high recovery efficiency of rare cells, simultaneously. However, the centrifugation-based approaches 17, 18 can only realize a high throughput while suffer low cell recovery efficiency. The micro/nano technique-based approaches 20-34, are promising to achieve a high cell recovery efficiency but suffer a low throughput, and thus fail to process large-volume clinical samples.

The BALF-based diagnosis should be more competitive in the diagnosis of peripheral lung cancer, because the BAL operation has access to the peripheral pulmonary lesions where the bronchoscopy can't reach 13. Bigger differences in sensitivity between the developed dual-layer PERFECT filter system and the cytocentrifuge, *i.e.* more prominent advantages of filtration, are anticipated in further multi-institutional clinical trials with recruitments of patients with small lung nodules under CT. The developed system will be promising to advance the cytological test of small nodules and fulfill the diagnosis of early lung cancer.

All BALF samples with three turbidity grades can be diagnosed effectively with the dual-layer PERFECT filter system. Figure 4C shows the sensitivities of all the three graded turbid samples with the PERFECT filter system are of no big difference, with 80.0% (4/5), 71.4% (5/7) and 87.5% (7/8) for 5 positive cases in clear group, 7 positive cases in slightly turbid group and 8 cases in extremely turbid group, respectively, being correctly diagnosed with the dual-layer PERFECT filter system. However, those values from the conventional cytocentrifuge are only 20.0% (1/5), 42.9% (3/7) and 62.5% (5/8), which are much lower than the dual-layer filtration method. For the clear and slightly turbid groups, filtration of the whole samples could finish smoothly without clogging, plus the high recovery efficiency of the PERFECT filter, therefore, higher sensitivities were achieved with the established dual-layer PERFECT filter system. While, the cytocentrifuge suffered cell loss from centrifugation and a limited operated sample volume as aforementioned, and thus presented relatively lower sensitivities. For the extremely turbid group, even though clogging happened and caused not the whole sample to be filtered (with unfiltered part discarded after an utmost filtration duration of 10 min), there still presented a higher sensitivity at 87.5% (7/8) with the dual-layer PERFECT filter system, compared to that (62.5% (5/8)) with the cytocentrifuge. The detailed reason for this merit will be further investigated. And also, pretreatment of the extremely turbid samples to fulfill higher-performance filtration and diagnosis is a worth noting direction to go in the further clinical trials. Besides, it seems that the sensitivity has a relationship with the turbidity grade. First, the sensitivities of clear, slightly turbid, extremely turbid samples are 45.5% (5/11), 53.9% (7/13) and 88.9% (8/9), respectively. On the other hand, the proportions of clear, slightly turbid, extremely turbid samples in the 20 correctly-diagnosed positive cases are 25.0% (5/20), 35.0% (7/20) and 40.0% (8/20), respectively. While the reason is not clear and we will try to investigate in future and hope to be able to report soon.

Moreover, the dual-layer PERFECT filter system is generally applicable to different types of lung cancer. As shown in Figure 4D, the 20 positive lung cancer cases in this trial include 8 cases of squamous cell carcinoma (SCC), 6 cases of adenocarcinoma (ADC), 3 cases of small cell lung cancer (SCLC) and 3 cases of metastatic cancer (metastasis from a non-lung primary carcinoma to the lung). The sensitivities with the proposed system are 75.0% (6/8), 83.3% (5/6), 100% (3/3) and 66.7% (2/3) for SCC, ADC, SCLC and metastatic cancer, respectively. However, those with the cytocentrifuge are only 50.0% (4/8), 33.3% (2/6), 66.7% (2/3) and 33.3% (1/3). It is easy to notice that there is the biggest difference between the dual-layer PERFECT filter system (83.3%) and the cytocentrifuge (33.3%) for the ADC type. This could be attributed to that ETCs from ADC patients usually appear in clusters 40, which are more easily enriched and recovered by Filter-3.

In the 16 positive cases diagnosed with the dual-layer PERFECT filter system, the ETCs were identified mainly on the upper-layer (Filter-3), although the recovery rate of A549s from spiking sample on the upper-layer (Filter-3) is as low as 6.7 ± 5.7% shown in Figure 2. The much higher positive detection rate could be attributed to that single A549 cells in the spiking samples is much smaller than the pore size of Filter-3 and passes through the Filter-3 easily, while ETCs in patient BALF samples usually appear in clusters or adhere to mucus and are more likely to be recovered on Filter-3. Typical images of cells/clusters recovered on the PERFECT filters and slides after centrifugation are displayed in comparison in Figure 4E-G. More ETCs (clusters) in more concentrated distributions are presented on the filters than on the slides, which can be attributed to both recovery and enrichment of target cells/clusters with the PERFECT filter system and its large operable volume of BALF samples. The established dual-layer filtration ensures most of small normal cells such as squamous cells, erythrocytes, lymphocytes, granulocytes, *etc.*, pass through the PERFECT filters, and thereby enrich the large, usually abnormal cells, besides macrophages. The large number and concentrated distribution of ETCs (clusters) will contribute to worth-expecting higher accuracy and diagnostic efficiency for the pathologists in clinical applications. Therefore, the PERFECT filter system can guarantee a clear view of cells on the PERFECT filters for an easy and rapid identification of the abnormal cells even after process of the whole BALF samples with a large volume and a large number of cells. On the contrary, the cytocentrifuge leads cells to pile up on the slides when the cell number is over the specific criteria in most clinical cases because of no selectivity of cells. To guarantee a monolayer of cells on slides, only a limited volume according to the pretested cell density in the sample, usually less than 10 mL, can be loaded into the Hettich sample cup based on the pre-measured cell concentration. The smaller operable sample volume is the leading reason for a lower cell recovery rate for the cytocentrifuge, compared to the PERFECT filter system. And the great dispersion of the rare ETCs increases the difficulty of identification and diagnostic efficiency for the pathologists. Overall, the developed dual-layer PERFECT filter system fulfills an efficient liquid biopsy of BALF targeted for lung cancer, and is well worth promoting through more clinical trials to advance its final practical applications.

### Feasibility of large-field scanning imaging for promising AI-assisted interpretation

**Figure 5:**
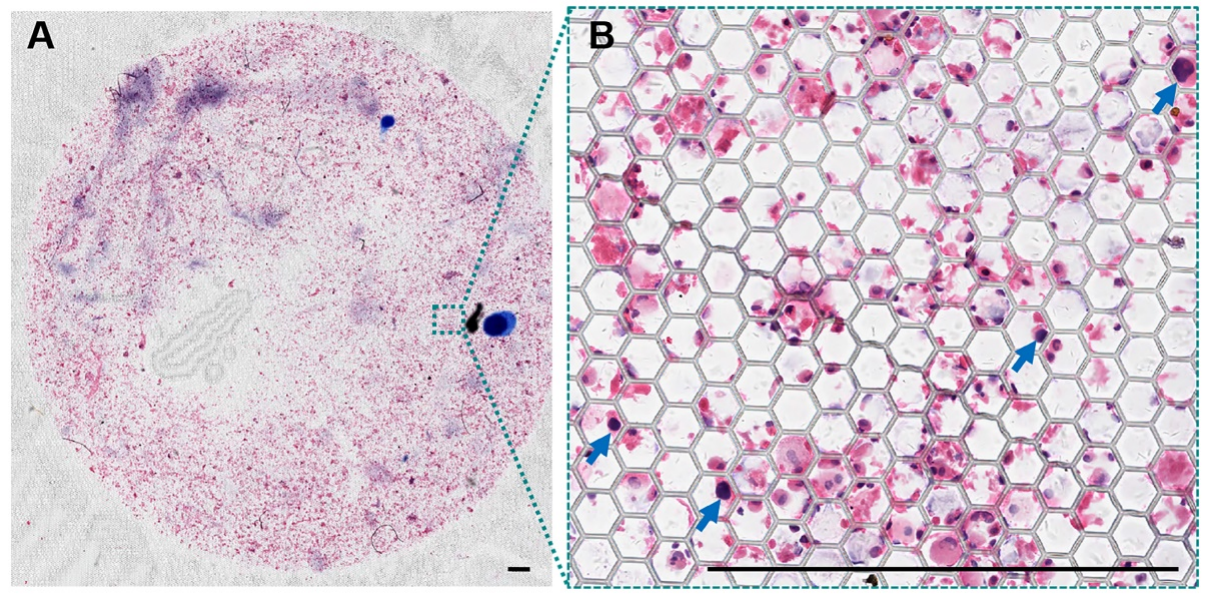
(A) A typical large-field scanning image of the whole Filter-3 after operation of BALF sample from patient #05. (B) The enlargement of the certain area with squamous carcinoma cells denoted with blue arrows. Scale bars represent 500 μm in A-B.

After the HE staining, PERFECT filters with recovered cells (clusters) were mounted by glass slides. The thickness of the filter is only about 10 μm, *i.e.* about the diameter of an individual cell. The filters with recovered cells after filtration are flat enough for direct reading, benefiting from a high mechanical strength 36. The above two advantages guarantee a high quality of scanning imaging over the whole effective filtration area (12 mm in diameter) of PERFECT filters, as shown in Figure 5. This facilitates a promising automatic interpretation of tumor cells with the rapid development of the artificial intelligence enabled image recognition technology. It's worthy of mentioning that the pathological interpretation based on machine learning might be a promising way to avoid the variations from different operators or physicians.

### Compatibility and feasibility for various downstream analyses

The HE staining-based cytology used in the present work is a routine approach in the department of pathology in hospitals, and can fulfill the preliminary interpretation. While, the diagnostic efficiency and accuracy largely depend on the experience of the pathologist to distinguish the tumor cells from lots of background cells. Therefore, further downstream analyses, such as immunocytochemistry (ICC), immunofluorescence (IF), fluorescent in situ hybridization (FISH), mass spectrum, sequencing, *etc.*, of the ETCs are important and worth doing to provide more information to fulfill further precise diagnosis and provide guide for personalized therapy. *In situ* assays (ICC, IF and FISH) are feasible with recovered cells staying on the PERFECT filters. Meanwhile, the mass spectrum and sequencing raise the requirement for cell release from the filters, which has been proved to be feasible.

Moreover, ETCs from BALF are thought to be able to reveal more information about the primary tumor site 41-43 than the present mainstream circulating biomarkers from peripheral blood, such as circulating tumor cells (CTCs) 44, 45 and circulating tumor DNA (ctDNA) 46, 47. In addition, there are increasing controversies about the discordancy between the blood-based liquid biopsy and tissue biopsy recently 48, and the genetic alterations have been also increasingly reported in controls, such as clonal hematopoiesis 49-51. These questionable points may hamper the specificity of non-invasive blood-based tests. Notably, results of proteomic and genomic analyses from several studies 52-55 support the conclusion that the BALF more than serum (blood-based) reflects the lung cancer. Above all, BALF is a promising source for liquid biopsy of lung cancer, and exfoliated cells are the valuable detection targets as intact cells containing both proteinic and genetic contents for comprehensive down-stream analyses.

## Conclusions

In this work, a high-efficiency liquid biopsy platform based on a dual-layer PERFECT filter system for rapid and sensitive separation and detection of ETCs from BALF samples is successfully developed. The ETC clusters and single ETCs have been efficiently recovered by the upper-layer filter with large micropores (feature size of 49.4 ± 0.5 μm) and the lower-layer filter with small micropores (9.1 ± 0.1 μm), respectively, followed by an *in-situ* HE staining for cytopathological identification. In the preliminary clinical trial including 20 lung cancer positive and 13 lung cancer negative cases, according to the histopathological diagnosis, a higher sensitivity (80.0%, 95% CI: 55.7%-93.4%) has been achieved with the established dual-layer PERFECT filter system, which is nearly double of that (45.0%, 95% CI: 23.8%-68.0%) obtained with the traditional cytocentrifuge, with *p*-value of 0.016 (McNemar test, two-tail). Both the dual-layer PERFECT filter system and cytocentrifuge present 100% specificity. Besides the high detection sensitivity and specificity, the developed system enables a short time-to-result, benefiting from the handling ability of large-volume (6-18 mL in this study) clinical BALF samples with complex contents at a high filtration throughput. The filtration of clinical BALF samples were finished within 3 min for 54.6% samples (18/33) and 10 min at most for the rest ones, with throughputs of 31.9 ± 17.3 mL/min and 3.2 ± 2.6 mL/min for clear and slightly turbid samples, respectively. Although the extremely turbid samples with filtration throughput of less than 0.5 mL/min, the PERFECT filter system still fulfill the diagnosis at a high sensitivity of 87.5% (7/8). Moreover, the handiness in system set-up, ease of operation and readiness for integration with various downstream analysis will facilitate the developed system to find wide applications in clinical practices.

The established dual-layer PERFECT system has demonstrated the detection of rare ETCs from BALF samples with a high detection sensitivity and promotes the clinical significance of liquid biopsy of BALF for lung cancer diagnosis. Thanks to the easier accessibility of BALF sampling than the tissue biopsy, the efficient and sensitive liquid biopsy of BALF is thought to be more advantageous in the peripheral lung cancer. The investigation of the dual-layer PERFECT filter system application in peripheral pulmonary lesions will be the key focus of the follow-up study, mainly with BALF samples from patients of small lung nodules under CT to be enrolled via a multi-institutional trial. That will be promising to advance the cytological test of nodules and fulfill the diagnosis of early lung cancer. Even more noteworthy is that the established dual-layer PERFECT filter system is feasible to be adjusted and find powerful uses in efficient separation and detection of rare tumor cells, fungi and bacteria from various samples such as blood, urine, sputum *etc.*, for different clinical applications to further expand the social and economic benefits.

## Figures and Tables

**Table 1:** Feature sizes and porosities of the PERFECT filters used in this work.

Case No.	Designed value (μm)		Practical value (μm)		Porosity (%)
	*d*	*s*	*d*	*s*	*p*
PERFECT Filter-1	10	4	9.1 ± 0.1	4.7 ± 0.2	46.7
PERFECT Filter-2	25	4	24.5 ± 0.1	4.5 ± 0.1	71.5
PERFECT Filter-3	50	4	49.4 ± 0.5	4.6 ± 0.1	83.8
PERFECT Filter-4	100	4	99.4 ± 0.5	4.5 ± 0.1	91.4

**Table 2:**
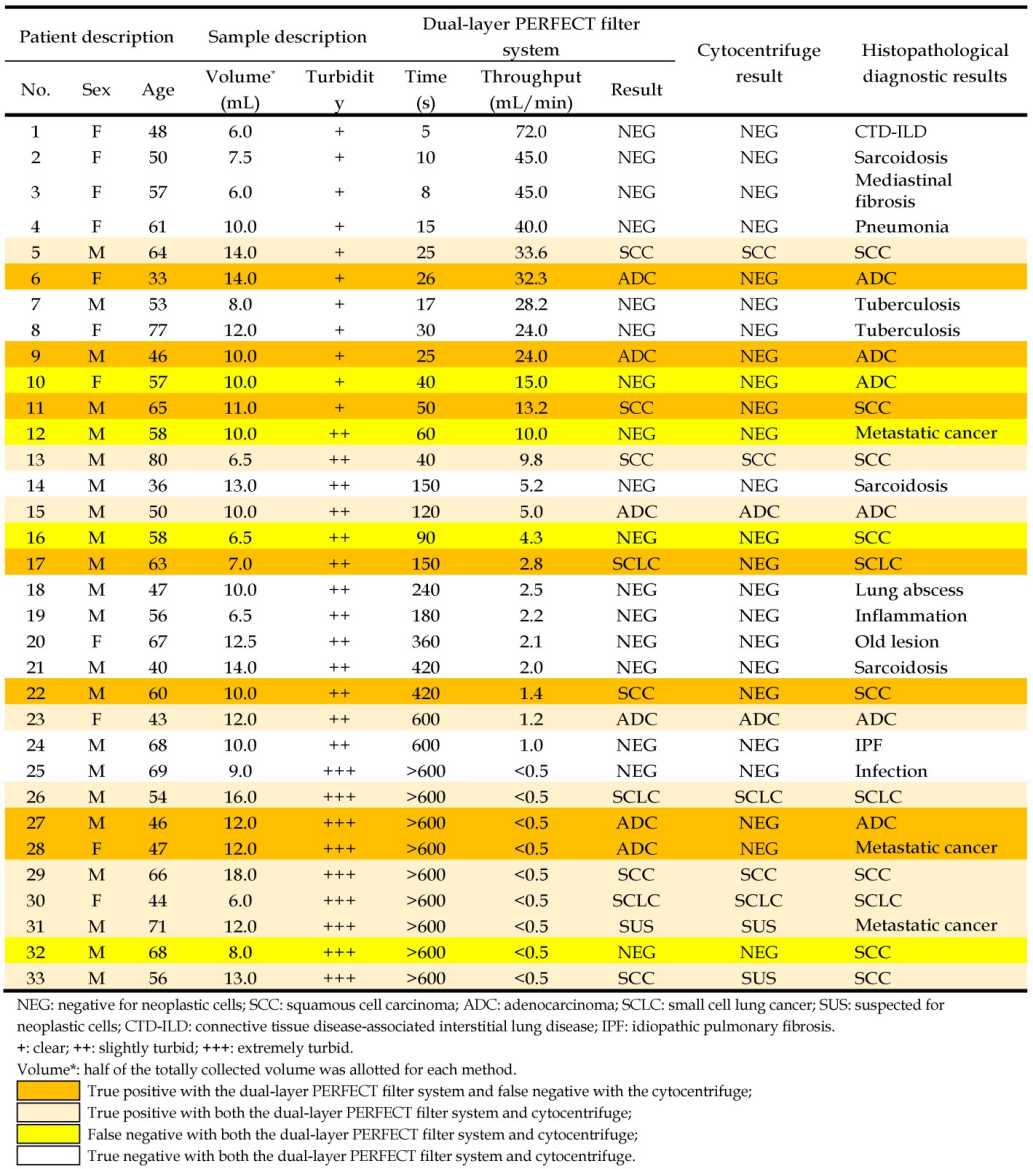
The diagnostic results of clinical BALF specimens by the dual-layer PERFECT filter system and the cytocentrifuge, compared to the definitive diagnosis from histopathological results.

## Abbreviations

PERFECT filter: precise, efficient, rapid, flexible, easy-to-operate, controllable and thin filter
ETC: exfoliated tumor cell
BALF: bronchoalveolar lavage fluid
CT: computerized tomography
PET: positron emission tomography
LDCT: low-dose computerized tomography
ATS: American Thoracic Society
ACCP: American College of Chest Physicians
SEM: scanning electron microscopy
BAL: bronchoalveolar lavage
HE staining: hematoxylin-eosin staining
NEG: negative for neoplastic cells
SCC: squamous cell carcinoma
ADC: adenocarcinoma
SCLC: small cell lung cancer
SUS: suspected for neoplastic cells
CTD-ILD: connective tissue disease-associated interstitial lung disease
IPF: idiopathic pulmonary fibrosis
AI: artificial intelligence
ICC: immunocytochemistry
IF: immunofluorescence
FISH: fluorescent in situ hybridization
CTC: circulating tumor cell
ctDNA: circulating tumor DNA
